# *In Vitro* Growth Inhibition, Caspase-Dependent Apoptosis, and S and G2/M Phase Arrest in Breast Cancer Cells Induced by Fluorine-Incorporated Gold I Compound, Ph3PAu[SC(OMe)=NC6H4F-3]

**DOI:** 10.1155/2022/7168210

**Published:** 2022-07-21

**Authors:** Richard Ming Chuan Yu, Gayathri Thevi Selvarajah, Geok Chin Tan, Yoke-Kqueen Cheah

**Affiliations:** ^1^Department of Biomedical Sciences, Faculty of Medicine and Health Sciences, Universiti Putra Malaysia, Serdang, Selangor, Malaysia; ^2^Department of Veterinary Clinical Studies, Faculty of Veterinary Medicine, Universiti Putra Malaysia, Serdang, Selangor, Malaysia; ^3^UPM-MAKNA Cancer Research Laboratory (CANRES), Institute of Bioscience, Universiti Putra Malaysia, Serdang, Selangor, Malaysia; ^4^Department of Pathology, Faculty of Medicine, Universiti Kebangsaan Malaysia, Bandar Tun Razak, Cheras, Malaysia

## Abstract

Gold-based anticancer compounds have been attracting increasing research interest due to their ability to kill cancer cells resistant to platinum-based compounds. Gold I- and gold III-based complexes have shown satisfactory anticancer activities. In this study, two new fluorine-incorporated gold (I) compounds such as Ph_3_PAu[SC(OMe)=NC_6_H_4_F-3] and DPPFeAu_2_[(SC(OMe)=NC_6_H_4_F-3)]_2_ were evaluated for their *in vitro* activities against human breast cancer cell lines, primary breast cancer cells, and breast cancer stem cells (parental breast cancer stem cells, BCSC-P, and breast cancer stem cells, BCSC). Assays for growth inhibition and cytotoxicity, including real-time cell analysis, were carried out to screen effective antibreast cancer compounds. In addition, further *in vitro* assays such as apoptosis, caspase 3/7 activity, and cell cycle analysis were performed to observe the action and mechanism of killing breast cancer cells by the selected gold I compound, Ph_3_PAu[SC(OMe)=NC_6_H_4_F-3]. The gold (I) compound, Ph_3_PAu[SC(OMe)=NC_6_H_4_F-3], showed low toxicity to H9c2 normal cells and significant growth inhibition in MDA-MB-231 and MCF-7 cells, primary breast cancer cells, and breast cancer stem cells (BCSC-P and BCSC). The IC_50_ doses of the gold (I) compound Ph_3_PAu[SC(OMe)=NC_6_H_4_F-3] against the breast cancer cell lines MDA-MB-231 and MCF-7 were approximately 6-fold lower than that of cisplatin (cis-diamineplatinum (II) dichloride, CDDP). Moreover, the compound Ph_3_PAu[SC(OMe)=NC_6_H_4_F-3] induced caspase 3/7-dependent apoptosis and cell cycle arrest at S and G2/M phases. Ph_3_PAu[SC(OMe)=NC_6_H_4_F-3], a gold (I) compound incorporated with fluorine, is a potential candidate for the treatment of breast cancer.

## 1. Introduction

Breast cancer is one of the deadliest diseases among women globally, with a very high incidence, 2.26 million (11.7%) of the total 19.29 million new cancer cases in year 2020 [1]. Moreover, approximately 10–15% of all breast cancer cases are triple-negative as they do not express receptors for estrogen, progesterone, and HER2 [2, 3] and are aggressive as well as difficult to treat as they do not respond to hormonal therapy. Despite increased survival rates in recent years due to advancements in technologies for early detection, significant efforts are being devoted to developing new and effective chemotherapeutic compounds against breast cancer. Recent findings have highlighted cancer stem cells as the root cause of cancers; these contribute to cancer recurrence within the first 5 years on average, after remission following treatment [4]. This reemergence of cancer stem cell theory has encouraged researchers to explore novel and effective metal-based compounds against cancer stem cells and advanced breast cancers.

Platinum derivatives have limitations in cancer treatment, considering their toxicity to the neurons and kidneys [5]. Furthermore, they show low effectiveness in cancers that are naturally resistant to cisplatin. The development of acquired resistance in patients previously treated with cisplatin also remains challenging. Overall, the existing metal-based anticancer compounds, including platinum-based compounds, are not very effective against triple-negative breast cancers, metastatic breast cancers, cisplatin-resistant breast cancers, and breast cancer stem cells (BCSC). The existing literature suggests that gold-based compounds are promising alternative agents that work on cisplatin-resistant breast cancer cells [6–8]. The anticancer activity of gold compounds was 8.7- to 20.8-fold higher than that of cisplatin [9]. Therefore, the need to explore more gold-based anticancer compounds to discover new drugs against breast cancer is evident.

Fluorine has unique physical and chemical properties such as being the lightest and the most reactive element among halogens. It can also be found as a trace element in our body parts especially in the teeth, bones, hair, blood, milk, and urine. Fluorine was successfully isolated in 1886 by Henri Moissan who won the Nobel Prize in 1906. Fludrocortisone was the first pharmaceutical item which incorporated fluorine atom to cortisol in 1954. The inclusion of fluorine into the drug design is known to amplify the pharmacological impact on bioactive molecules by increasing the biological activity, enhancing hydrogen bonding interactions and binding affinity to target proteins, and promoting chemical and metabolic stability [10, 11].

Therefore, in this study, the anticancer properties of the novel fluorine-incorporated gold (I) compounds such as triphenylphosphanegold (I) O-methyl-N-3-fluorophenyl thiocarbamate: Ph_3_PAu[SC(OMe)=NC_6_H_4_F-3] (compound 3F1), Bis (diphenylphosphinoferrocene) di gold I O-methyl-N-(3-fluorophenyl) thiocarbamate): DPPFeAu_2_[(SC(OMe)=NC_6_H_4_F-3)]_2_ (compound 3F3), and their ligand LH (compound 3FL) synthesized by Yeo and colleagues [12, 13] were evaluated in breast cancer cells. The compounds were tested for growth inhibition, apoptosis induction, and cell cycle arrest in human breast cancer cell lines such as MDA-MB-231 (triple-negative breast cancer cells) and MCF-7 (estrogen receptor-positive breast cancer cells), primary breast cancer cells (in-house cultured), BCSC, and parental BCSC (BCSC-P). Based on the results, the new gold (I) compound, Ph_3_PAu[SC(OMe)=NC_6_H_4_F-3], showed promising anticancer properties against different types of breast cancers *in vitro*, in terms of growth inhibition, apoptosis induction, and cell cycle arrest.

## 2. Materials and Methods

### 2.1. Cell Cultures

Triple-negative breast cancer cells MDA-MB-231 (ATCC HTB-26), estrogen receptor-positive breast cancer cells MCF-7 (ATCC HTB22), and normal myoblast cells H9c2 (ATCC CRL-1446) were sourced from the American Type Culture Collection (ATCC). BCSC-P (Celprogen 36102-29P) and BCSC (Celprogen 36102-29) were purchased from Celprogen. Primary breast cancer cells were cultured in our laboratory directly from biopsy tissue samples provided by Professor. Dr. Tan Geok Chin, Hospital UKM (ethical approval: UKM 1.5.3.5/244/FF-2015-182).

MDA-MB-231, MCF-7, and H9c2 cells were cultured and maintained in high-glucose Dulbecco's modified Eagle's medium (DMEM) with 10% FBS (heat-inactivated) and 1% pen-strep. Cells were incubated at 37°C in a humidified incubator (CCL-170B-8, ESCO, Singapore) containing 5% CO_2_ and 95% air.

BCSC-P (Celprogen 36102-29P, USA) and BCSC (Celprogen 36102-29, USA) were cultured in complete medium (Celprogen M36102-29U, USA) and were incubated overnight at 37°C in a humidified incubator (Innova CO14, New Brunswick Scientific, USA) with premixed gas at a ratio of 5% CO_2_, 5% O_2_, and 90% N_2_. The medium was changed once every 2 to 3 days until the cells were 80–90% confluent. Cells were subcultured until the third passage for further experiments.

Primary breast cancer cells were cultured from a patient tissue sample transported in iced cold L15 medium supplemented with 10% FBS and 1% pen-strep. Blood, blood clots, and fat were removed from the tissue sample and washed five times with sterile 1X PBS followed by dissociation it in 2 mL of 0.25% trypsin-EDTA (Gibco 15400-054, USA) and placed it in a conventional cell culture incubator (CCL-050B-8, ESCO, Singapore) for 4 hr. Mechanical dissociation was also done for every 1 hr. Dissociated tissue samples were then cultured in DMEM high glucose supplemented with 10% FBS (heat-inactivated) and 1% pen-strep and were incubated overnight at 37°C in a humidified cell culture incubator with 5% CO_2_ and 95% air. After six passages, the cells were subcultured with serum-free DMEM/F12 (Nacalai 0846095, Japan) to inhibit fibroblast growth and promote epithelial cell growth. Primary breast cancer epithelial cells were subcultured until passage 16.

### 2.2. Gold Compounds

Triphenylphosphanegold (I) O-methyl-N-3-fluorophenyl thiocarbamate (compound 3F1) also known as [(Z)-N-(3-Fluorophenyl)-O-methylthiocarbamato-кS](triphenylphosphine-кP) gold(I) and Bis (diphenylphosphinoferrocene) di gold (I) O-methyl-N-(3-fluorophenyl) thiocarbamate (compound 3F3) which is also known as (*μ*_2-_1,1′-bis (diphenylphosphino) ferrocene-K^2^ P,P′)-bis[(Z)N-(3-fluorophenyl)-O-methylthiocarbamato-S] digold (I) chloroform solvate and their ligand LH (compound 3FL) used for compound 3F1 and compound 3F3 were synthesized by Yeo et al. and collected them for this study after confirmation of their purity was tested by using nuclear magnetic resonance [11, 12].

### 2.3. Cell Viability and Cytotoxicity Assays

The cytotoxicity of each compound against breast cancer cells was measured with a conventional MTT assay [10] followed by a real-time cell analyzer, iCelligence [14, 15]. MCF-7, MDA-MB-231, and primary breast cancer (BCA) cells were seeded at 2.5 × 10^4^ cells, and H9c2 cells were seeded at 3 × 10^4^ cells/well of a flat-bottom 96-well plate each (SPL Life Sciences, Korea), in 100 *μ*L of complete DMEM high glucose and incubated at 37°C in a humidifier incubator supplied with 5% CO_2_ and 95% air for 24 h. BCSC-P and BCSC were seeded at 2 × 10^4^ cells/well of flat-bottom 96-well plates (Celprogen E36102-29P-96 and Celprogen U36102-29-96), respectively, in 100 *μ*L of complete culture medium (Celprogen, M36102-29US). All cells were in the exponential growth phase. Plates seeded with stem cells were incubated at 37°C in a minihumidified incubator (Innova CO14, New Brunswick Scientific, USA) supplied with premixed gas (5% CO_2_, 5% O_2_, and 90% N_2_) for 24 h.

The cells were then treated with various concentrations of gold compounds (80 *μ*M, 40 *μ*M, 20 *μ*M, 10 *μ*M, 5 *μ*M, and 2.5 *μ*M). As negative controls, cells were treated with 0.1% DMSO. Cells in positive control wells were treated with freshly prepared IC_50_ of cisplatin (Sigma P4394, USA). Twenty microliters each of MTT (5 mg/mL) was added to each well and incubated for 3 h. To dissolve the formazan crystals, medium containing MTT was aspirated gently followed by adding 100 *μ*L DMSO into each well of 96-well plate.

For BCSC-P and BCSC, 10 *μ*L of MA dye (Biolog 74351, USA) was added to each well. The absorbance of the plates with MA dye was read directly after 3 h incubation without removing the medium-plus dye mixture and adding the solvent DMSO because the MA dye forms soluble formazan crystals and develops color directly.

The plates were analyzed using a microplate reader (MicroStation ELX808BLG, Biolog, USA) at 590 nm with a reference wavelength of 650 nm to measure the absorbance value for each well [13, 16]. The percentage of cell viability was calculated using the formula below [17], and IC_50_ of the gold compounds against breast cancer cells was determined using GraphPad Prism 7.

(1) $$\begin{array}{c} \text{Cell viability}\%=\frac{\text{A}590\text{ nm }\left(\text{Treated}\right)-\left(\text{Blank}\right)}{\text{A}590\text{ nm }\left(\text{Control}\right)-\left(\text{Blank}\right)}\times 100, \end{array}$$

Where;

Treated:medium, cells and compound

Control:medium, cells, and 0.1% DMSO

Blank:medium only

Further analysis of cell viability was done by real-time cell analysis (RTCA) experiments for MCF-7, MDA-MB-231, and H9c2 cells treated with the compound 3F1. Briefly, 8-well E- plate (L8, ACEA biosciences, USA) was primed with 150 *μ*L of complete DMEM high glucose for 20 min, and the background electrical impedance was measured for 1 min. In total, 2.0 × 10^4^ cells/well in 350 *μ*L of complete DMEM high glucose were then seeded into the E-plate, which contained 150 *μ*L/well of complete DMEM high glucose. The E-plate was placed on the RTCA instrument and incubated for 24 h at 37°C in a humidified incubator supplied with 5% CO_2_ and 95% air. Cells in the E-plate were then treated with the selected gold compound, 3F1, in duplicate. Cells in the negative control well and positive control well were treated with DMSO (0.1%) and cisplatin at its IC_50_ (Sigma P4394, USA), respectively. The E-plate was again placed on the RTCA instrument for 24 h at 37°C in a humidified incubator supplied with 5% CO_2_ and 95% air for monitoring the cells in a real-time manner. Data analysis was performed using the RTCA analysis software version 1.0 for determining the normalized cell index at the start of the treatment time-point for time-dependent IC_50_.

### 2.4. Acridine Orange and Propidium Iodide Dual Fluorescence Cell Viability Assay

MDA-MB-231 and MCF-7 cells were seeded at a density of 4 × 10^5^ cells/mL of complete DMEM high glucose to T25 flasks and incubated for 24 h at 37°C in a humidified incubator (CCL-170B-8, ESCO, Singapore) supplied with 5% CO_2_ and 95% air for the acridine orange and propidium iodide (AO/PI) cell viability assay using Luna-FL [18, 19]. Cells were treated with the respective IC_50_ dose of 3F1 (8.5 *μ*M for MDA-MB-231 and 6.5 *μ*M for MCF-7) or 0.1% DMSO (control) and incubated. Total 10 *μ*L of the cell suspension stained with AO/PI dye (Logos Biosystems, F23001, Korea) was loaded onto a PhotonSlide cell counting chamber (Logos Biosystems, L12005, Korea). In Luna-FL AO/PO assay, fluorescence green or yellowish-green captured images are recognized as viable cells, and red fluorescence images are recorded as nonviable cells.

### 2.5. Annexin V and Dead Cell Assay

MDA-MB-231 and MCF-7 cells were seeded at 4 × 10^5^ cells/mL into T25 flasks and incubated for 24 h. The cells were then treated with the respective IC_50_ dose of the gold compound, 3F1, and incubated for a further 24 h. Treated cells were stained with MUSE Annexin V-PE and dead cell reagent 7-AAD according to the manufacturer's instructions (Merck-Millipore, USA) [20–22]. The MUSE cell analyzer was used to detect the stained cells, which were segregated into four quadrants of a scattered plot to distinguish between live cells, early apoptotic cells, late apoptotic cells, and necrotic cells. Three independent experiments were carried out.

### 2.6. Caspase-3/-7 Activity Assay

MDA-MB-231 and MCF-7 cells were seeded at a density of 5 × 10^5^ cells/mL with complete DMEM high glucose (Nacalai, Japan) in T25 flasks and incubated for 24 h at 37°C in a humidified incubator (CCL-170B-8, ESCO, Singapore) with 5% CO_2_ and 95% air. Cells were treated with 0.1% DMSO (control) or with the IC_50_ dose of the 3F1 compound. Single-cell suspension at the recommended density between 1 × 10^5^ and 5 × 10^6^ cells/mL in 1X assay buffer BA was prepared. Staining the cells with DNA-binding dye coupled with DEVD peptide substrate was performed according to the manufacturer's instructions [23, 24]. The results were analyzed using MUSE analysis software version 1.5. Stained cells were distinguished into four quadrants in a scattered plot.

### 2.7. Cell Cycle Analysis Assay

MUSE cell analysis assay kit (Millipore, USA) that contains PI and RNase A premixed reagent was used for cell cycle analysis. MDA-MB-231 and MCF-7 cells were seeded at a density of 5 × 10^5^ cells/mL in complete DMEM high glucose (Nacalai, Japan) in T25 flasks (SPL Life Sciences, Korea) and incubated for 24 h at 37°C in a humidified incubator (ESCO, Singapore) with 5% CO_2_ and 95% air. Cells were treated with different doses of 3F1: e.g., IC_50_, lower than IC_50_, higher than the IC_50_ to evaluate whether the compound 3F1 consistently affected the cell cycle of these breast cancer cells. Treated and control cells were incubated for 24 h at 37°C in a humidified incubator supplied with 5% CO_2_ and 95% air followed by fixation and staining of the cells by manufacturer instructions of the kit. Analysis was done using the MUSE cell analyzer (Merck-Millipore, USA) followed by ModFit LT version 5 to generate the histograms and the percentage of cells in each cell cycle phase.

### 2.8. Statistical Analysis

Data presented were mean ± SD from three independent experiments with at least duplicate or triplicate. IC_50_ and statistical comparisons were calculated using GraphPad Prism software version 7 (GraphPad, San Diego, CA, USA). Statistical significance was analyzed by one-way ANOVA and a post hoc Dunnett's multiple comparison test, ^∗∗∗^*p* < 0.001, ^∗∗^*p* < 0.01, and ^∗^*p* < 0.05 were considered as significantly different from control.

## 3. Results

### 3.1. Effects of Gold Compounds on Breast Cancer Cell Viability

**Figure 1:**
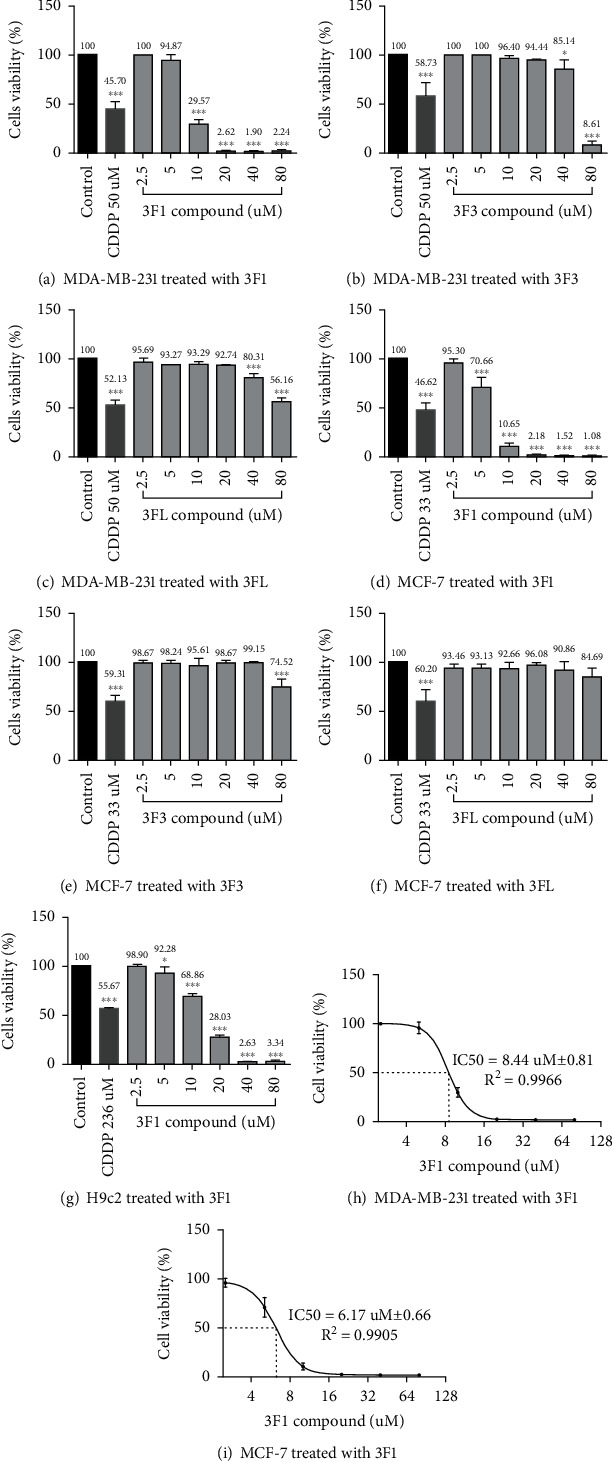
Viability percentage of cells by MTT assays. (a–c) MDA-MB-231 cells treated by the gold compound 3F1, 3F3, and 3FL. (d–f) MCF-7 cells treated by the gold compound 3F1, 3F3, and 3FL. (g) H9c2 cells treated by the gold compound 3F1. (h, i) Dose-response curves to determine the IC_50_ of the compound 3F1. Cells were treated with various concentrations of 3F1, 3F3, and 3FL compound for 24 h. Cells in the control wells were treated with 0.1% DMSO. MDA-MB-231, MCF-7, and H9c2 cells in positive control wells were treated with cisplatin (cis-diamineplatinum (II) dichloride, CDDP) at 50 *μ*M, 33 *μ*M, and 236 *μ*M, respectively. Data are presented as the mean ± S.D. of three independent experiments performed in triplicate. Statistical significance was analyzed by one-way ANOVA and a post hoc Dunnett's multiple comparison test. ^∗∗∗^*p* < 0.001 was considered significantly different from the control.

**Figure 2:**
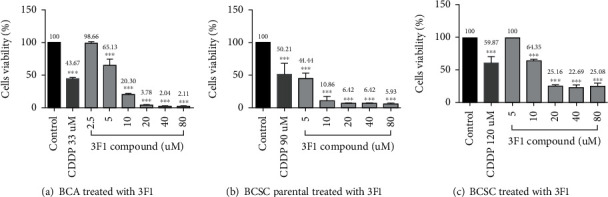
Viability percentage of cells by MTT assays. (a) Primary breast cancer cells. (b) Parental breast cancer stem cells. (c) Breast cancer stem cells were treated with various concentrations of compound 3F1 for 24 h. Cells in control wells were treated with 0.1% DMSO. Cells in the positive control wells were treated with CDDP (cisplatin) at 33 *μ*M, 90 *μ*M, and 120 *μ*M, respectively. Data are presented as the mean ± S.D. of three independent experiments performed in triplicate. Statistical significance was analyzed by one-way ANOVA and a post hoc Dunnett's multiple comparison test. ^∗∗∗^*p* < 0.001 and ^∗^*p* < 0.05 were considered as significantly different from control.

The effects of the compounds 3F1, 3F3, and the ligand LH (3FL) on the triple-negative breast cancer cells (MDA-MB-231), estrogen receptor-positive breast cancer cells (MCF-7), and normal cardiomyoblasts (H9c2) were examined using conventional MTT assays. The compound 3F1 showed significant inhibition of viability in MDA-MB-231 and MCF-7 cells at IC_50_ doses, 8.435 ± 0.809 *μ*M and 6.174 ± 0.660 *μ*M, respectively, and showed less toxicity to H9c2 normal cardiomyoblasts, with an IC_50_ of 13.670 ± 1.580 *μ*M. Both 3F3 and LH did not demonstrate the desired anticancer effects against either MDA-MB-231 or MCF-7 cells as they showed an IC_50_ greater than 60 *μ*M. Among the three compounds tested (3F1, 3F3, and 3FL), 3F1 showed effective in vitro anticancer activity (Figure 1). In addition to breast cancer cell lines MDA-MB-231 and MCF-7, MTT cells viability assays were carried out against other breast cancer cells such as BCA, BCSC-P, and BCSC to determine IC_50_ of the compound 3F1 (Figure 2). The compound 3F1 demonstrated significant growth inhibition of BCA, BCSC-P, and BCSC, and IC_50_ doses against these breast cancer cells were found to be less than the IC_50_ dose for H9c2 normal cardiomyoblasts too (Table 1).

**Figure 3:**
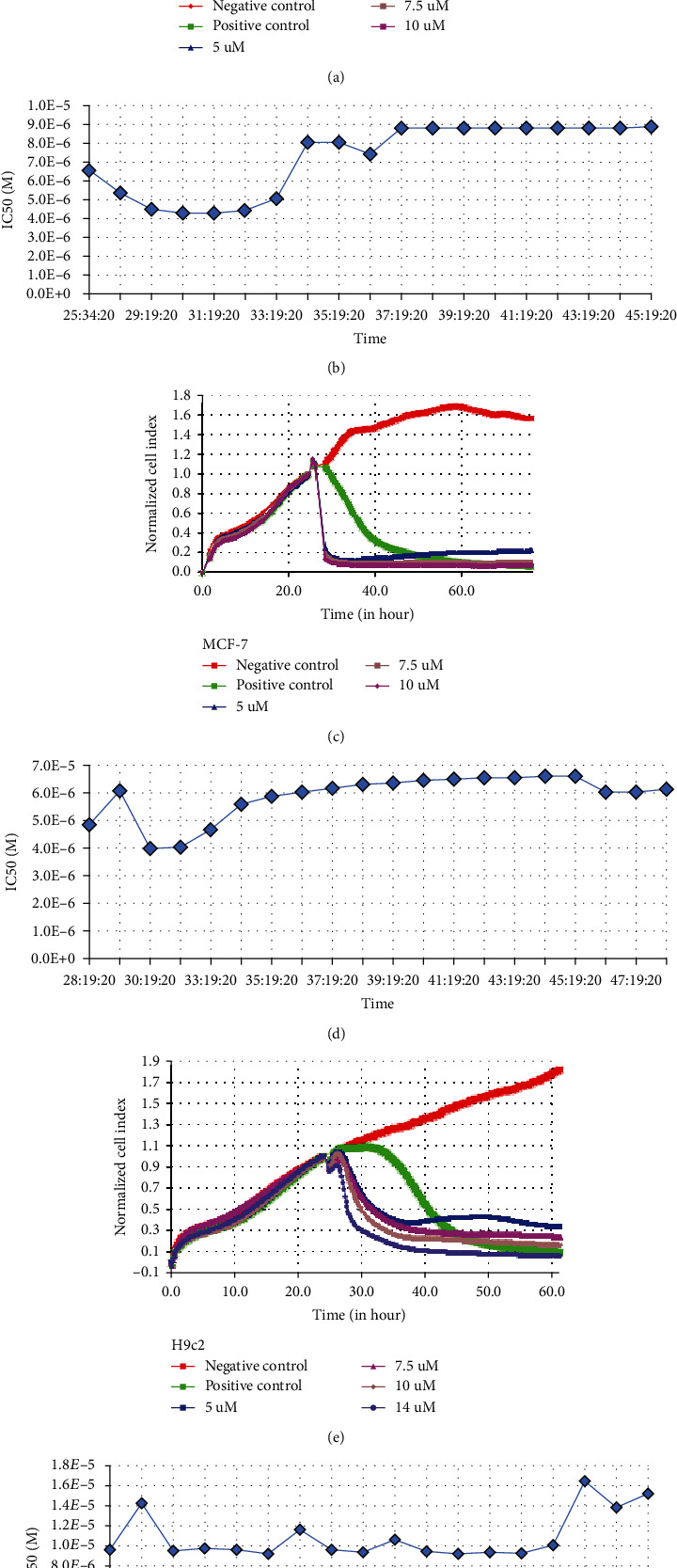
Cell viability of 3F1 determined by real-time cell analysis. (a, c, e) Normalized cell index. (b, d, f) Real-time-dependent IC_50_ of compound 3F1 against MDA-MB-231, MCF-7, and H9c2 cells. MDA-MB-231, MCF-7, and H9c2 cells were treated with DMSO for negative control, treated with CDDP (positive control), or the indicated concentrations of compound 3F1 for 24 h in E-plates. Data are presented as the mean ± S.D. of two independent experiments performed in duplicate.

In addition to the endpoint IC_50_ generated by MTT assay, RTCA assays were also performed to determine IC_50_ of the compound 3F1, and it was found that both endpoint IC_50_ doses generated from MTT and RTCA were comparable (Figure 3 and Table 2).

**Figure 4:**
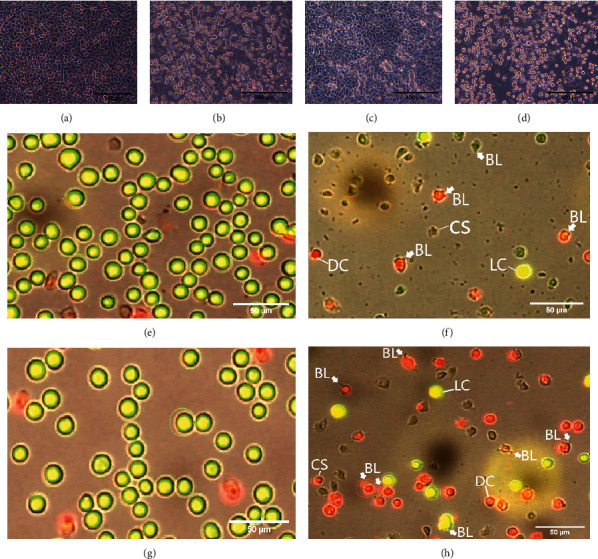
Breast cancer cells under the inverted microscopic view and on the screen view of Luna-FL. Microscopic view for MDA-MB-231 (control: (a), treated: (b)), MCF-7 (control: (c), treated: (d)). Luna-FL on-screen view of AO/PI-stained MDA-MB-231 (control: (e), treated: (f)), MCF-7 (control: (g), treated: (h)). LC: live cells; DC: dead cells; LB: membrane blebbing; CS: cell shrinkage. Cells were treated with the IC_50_ dose (8.5 *μ*M for MDA-MB-231 and 6.5 *μ*M for MCF-7) of compound 3F1 for the test samples or 0.1% DMSO for the control samples for 24 h. AO/PI-stained cells were viewed and counted on the Luna-FL (Logos Biosystems, Korea).

AO/PI cell viability assays were used to check further whether the identified IC_50_ dose (MTT and RTCA) of the novel gold-based compound 3F1 inhibited the growth of breast cancer cells before further tests were carried out. The AO/PI dual fluorescent dye was used to stain MDA-MB-231 and MCF-7 cells treated with the IC_50_ dose of compound 3F1 (8.5 *μ*M for MDA-MB-231 and 6.5 *μ*M for MCF-7) or 0.1% DMSO (control) for 24 h. The images and percentages of live green fluorescent cells and dead red fluorescent cells generated by the Luna-FL system (Logos Biosystems, Korea) are shown in Figure 4 and Table 3, respectively. The Luna AO/PI cell viability assay showed 29.29% and 64.54% viable cells in the treated MDA-MB-231 and MCF-7 cells, respectively.

### 3.2. Apoptosis Induction in Breast Cancer Cell Lines by Compound 3F1

**Figure 5:**
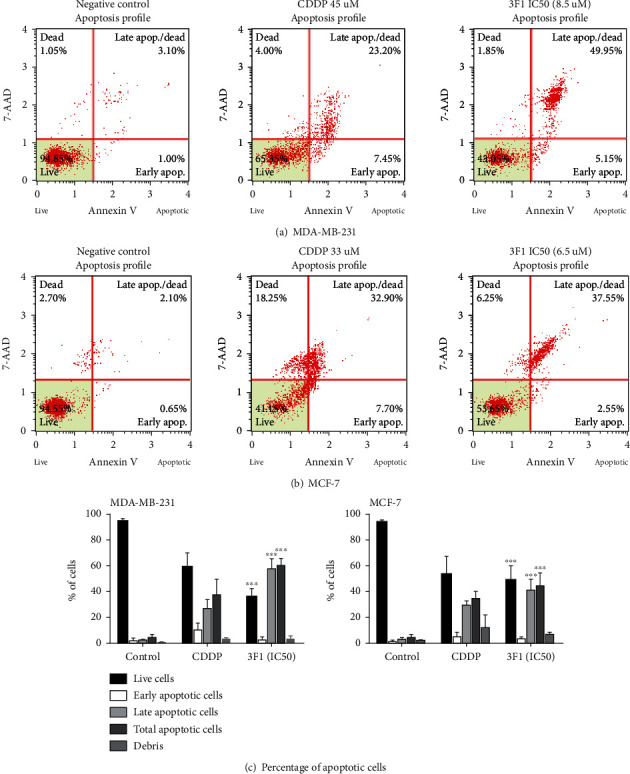
MUSE Annexin V and dead cell assay for detecting apoptosis induction. MDA-MB-231 (a) and MCF-7 (b) cells were treated for 24 h with the respective IC_50_ dose of the novel gold-based compound 3F1 or cisplatin (CDDP) for the positive control samples or with 0.1% DMSO for the negative controls. The representative scattered plot showed early apoptotic cells and late apoptotic cells in the lower and upper right quadrants. In contrast, live cells and necrotic cells were detected in the lower and upper left quadrant, respectively. Percentage of apoptotic cells (c) represented as the mean ± S.D. of three independent experiments. Statistical significance was analyzed by two-way ANOVA and a post hoc Tukey's multiple comparison test. ^∗∗∗^*p* < 0.001 was considered significantly different.

Annexin V, a cellular protein found in eukaryotic cells, has a high affinity to phosphatidylserine (PS), a phospholipid found on the inner surface or the cytoplasmic face of the cell membrane [25]. PS molecules are translocated to the outer membrane surface when cells undergo the apoptotic process [26]. Translocated PS molecules present in early and late apoptotic cells were bound by Annexin V-PE. Dead cells were detected by 7-AAD. Therefore, cells were distinguished into four groups in a scattered plot: viable cells (Annexin V^−^/7-AAD^−^) at lower left quadrant, early apoptotic cells (Annexin V^+^/7-AAD^−^) in the lower right quadrant, late apoptotic cells together with secondary necrotic cells (Annexin V^+^/7-AAD^+^) in the upper right quadrant, and necrotic cells without going through the apoptotic pathway (Annexin V^−^/7-AAD^+^) at the upper left quadrant. Breast cancer cell death via apoptotic pathway was detected by treating the breast cancer cells with the respective IC_50_ dose of 3F1 compound for 24 h and analyzed them using the MUSE Annexin V and dead cell kit (Merck-Millipore, USA) on the MUSE flow cytometer (Figure 5). Breast cancer cells treated with compound 3F1 showed an increasing average total apoptotic cell population (early apoptotic plus late apoptotic cells) compared with the control or the cells treated with CDDP (MDA-MB-231: control 4.60 ± 2.19%, CDDP 37.62 ± 12.07%, and 3F1 60.44 ± 5.55%; MCF-7: control 4.52 ± 2.53%, CDDP 34.64 ± 5.35%, 3F1 44.49 ± 9.85%).

### 3.3. Involvement of Caspase-3/-7 in Breast Cancer Cell Apoptosis Induced by Compound 3F1

**Figure 6:**
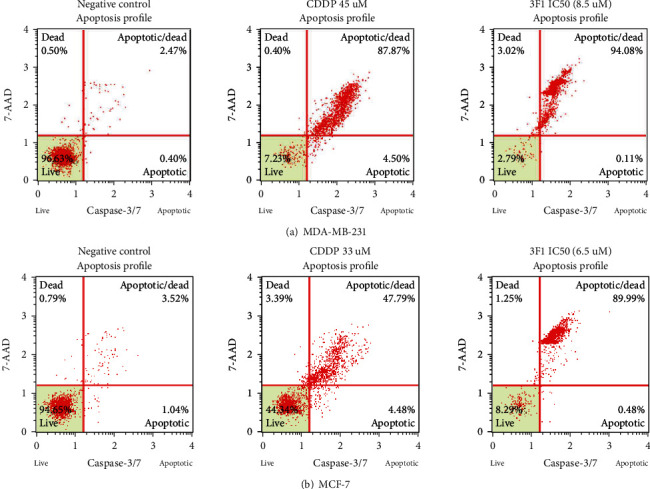
Caspase-3/-7-dependent apoptosis in cells treated with compound 3F1 were detected using a MUSE flow cytometer. MDA-MB-231 (a) and MCF-7 (b) cells were treated for 24 h with the respective IC_50_ dose of the novel gold-based compound 3F1 or with the respective IC_50_ dose of cisplatin (CDDP) for positive control samples or with 0.1% DMSO for the negative controls. Apoptotic cells with activated caspase-3/-7 are plotted in the upper and lower right quadrants. Viable cells are plotted in the left lower quadrant, whereas necrotic cells are shown in the left upper quadrant of the representative scattered plot.

Induction of the apoptosis pathway by compound 3F1 was tested by the detection of caspase-3/-7 activity. The bright fluorescent signals from caspase-dependent apoptotic cells were detected after cleavage of the peptide DEVD from the dye by activated caspase-3/-7 in the cells. The dye-free from DEVD is enabled to bind the DNA of apoptotic cells. MDA-MB-231 and MCF-7 cells treated with compound 3F1 for 24 h were apoptotic via caspase-dependent pathway (Figure 6). The total apoptotic cell population showed caspase-3/-7 activity was high in both MDA-MB-231 and MCF-7 cells compared to that of control samples.

### 3.4. Induction of Cell Cycle Arrest at the S and G2/M Phases in Breast Cancer Cells by Compound 3F1

**Figure 7:**
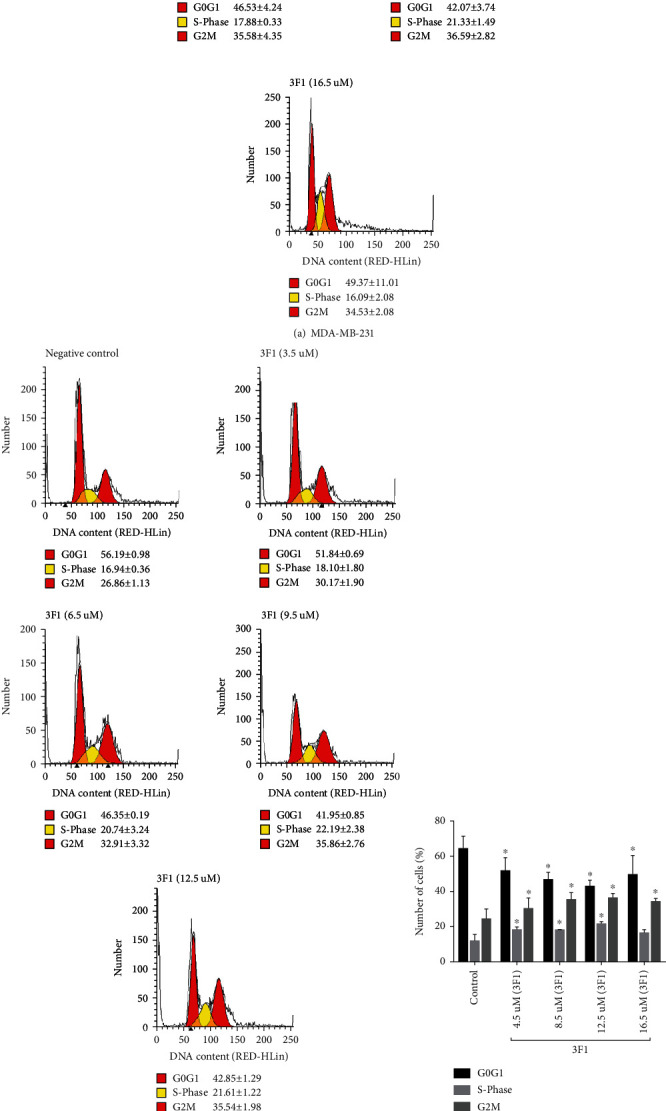
Percentage of cells at G0G1, S, and G2M phase. MDA-MB-231 (a, c) and MCF-7 (b, d) cells were treated with the indicated doses of compound 3F1 for 24 h. Cells in the negative control samples were treated with 0.1% DMSO. The cells were then fixed with ice-cold 70% ethanol and stained with propidium iodide (PI) from the MUSE cell cycle kit for MUSE flow cytometry analyses. S and G2/M phase cell population was consistently high while G0G1 cell population was consistently low in the treated breast cancer cells compared to those of nontreated cells. Data are presented as the mean ± SD of three independent experiments.

The compound 3F1 induced cell cycle arrest at both S and G2/M phases compared to the untreated control samples. MDA-MB-231 and MCF-7 cells were treated with compound 3F1 for 24 h, and cell cycle distribution was determined using MUSE cell cycle assay kits (Merck-Millipore, USA). MDA-MB-231 and MCF-7 cells were treated with 3 different doses such as IC_50_ dose, each dose lower than or higher than IC_50_ dose of compound 3F1 to check if they showed a consistent pattern of the compound activity on the cell cycle (Figure 7).

## 4. Discussion

In this study, *in vitro* anticancer activities of the gold (I) compound incorporated with fluorine, Ph_3_PAu[SC(OMe)=NC_6_H_4_F-3], have been demonstrated in terms of inhibiting the growth of MCF-7, MDA-MB-231, primary breast cancer cells, and BCSC, inducing apoptosis and arresting the cells cycle at both S and G2/M phase of MCF-7 and MDA-MB-231. A compound demonstrating *in vitro* antitumor activity at less than 10 *μ*M is considered an active hit-to-lead compound in drug discovery [27, 28]. In our screening, the compound 3F1 was considered a hit-to-lead compound as the respective IC_50_ dose against breast cancer cells such as MDA-MB-231 (8.435 *μ*M ± 0.809), MCF-7 (6.174 *μ*M ± 0.660), BCA (5.770 *μ*M ± 0.907), and BCSC-P (4.522 *μ*M ± 0.537) was less than 10 *μ*M, though this was not observed for BCSC, which showed an IC_50_ of 10.170 *μ*M ± 0.417. This finding is aligned with the common clinical perspectives wherein cancer stem cells are difficult to kill and have the potential for drug resistance [29]. Antitumor compounds usually target fast-growing tumor masses compared to slow dividing cells such as normal cells and stem cells, including cancer stem cells [30–32]. In other words, cells with high metabolic rates are killed faster than those with normal or slow metabolic rates. An accelerated metabolic rate of glucose is observed in cancer cells via aerobic glycolysis, wherein pyruvate is converted to lactate, also known as the Warburg effect [33]. We found that the IC_50_ dose of compound 3F1 was approximately sixfold lower than that of cisplatin (cis-diamineplatinum (II) dichloride). Alternatively, the novel gold-based compound 3F1 demonstrated sixfold stronger anticancer properties than cisplatin. In our study, endpoint IC_50_ does of compound 3F1 from MTT assay was confirmed by iCelligence RTCA. RTCA is one of the latest technologies useful for *in vitro* experiments to monitor cell proliferation, viability, morphology, and migration in a real-time manner. RTCA data are recorded based on the electrical impedance changes in gold electrodes due to the attachment of live cells [14]. Several RTCA assays have been previously conducted using the same types of breast cancer cells. For example, the effect of 6-bromo-indirubin on MDA-MB-231, the cytotoxicity of platinum II complexes against MDA-MB-231 and MCF-7, and the long-term effects of aluminum against MDA-MB-231 were determined by RTCA analyses [25–27]. The cell viability and dose-dependent cytotoxicity data from RTCA were in agreement with the MTT results [34]. From this study, we confirmed that endpoint IC_50_ doses were comparable between MTT assays and RTCA assays.

Apoptosis of breast cancer cells treated by compound 3F1 was detected by Annexin V assay. Following 24 h treatment with the compound, an increased percentage of the total apoptotic cell population was observed in MDA-MB-231 (60.44 ± 5.55%) and MCF-7 (44.49 ± 9.85%). Anticancer agents that induce apoptosis are highly desirable because malignant cells bypass apoptosis for their survival [35]. Induction of apoptosis is preferable over necrosis in therapeutics, including cancer treatments, as apoptosis requires a shorter period in the process of cell death [36]. Therefore, the novel gold compound 3F1, which induces apoptosis in breast cancer cells, is a potentially efficient chemotherapeutic agent against breast cancer. Apoptosis of breast cancer cells induced by compound 3F1 was further analyzed to check if it was involved in caspase activity. Caspases in human are divided into three subfamilies such as ICE-like protease (caspase-1, -4, -5, and -13), CED-3 protease (caspase-3, -6, -7, -8, -9, and -10) and the orphan caspase, caspase-2. Apoptosis can be executed through either caspase-dependent or caspase-independent pathways. Caspase-independent pathways have not been examined until recently [37]. To date, only two major caspase-dependent apoptosis pathways, i.e., the intrinsic pathway (via mitochondria) and extrinsic pathway (via cell surface death receptors), have been reported. The endoproteases, caspase-3/-7/-6, also named effectors or executioner caspases, are activated intracellularly by the initiators as a downstream cascade in response to apoptotic signals and lead cellular breakdown eventually. Both the extrinsic and intrinsic pathways of apoptosis must finally undergo activation of the executioner caspase-3/-7 and -6 [38, 39]. The intrinsic apoptotic pathway signals originate within the cells from DNA damage, cellular stress due to growth factor insufficiency, and cytoskeletal disruption. Once DNA damage occurs in cells, signaling molecules such as ATM-Chk2 and ATR-Chk1 activate p53 (tumor suppressor protein) and other oncogenes. Activated p53 blocks the cells from progressing to further stages in the cell cycle. Simultaneously, P53 starts to recruit the apoptosis regulator protein, BAX, which eventually induced pores in the mitochondria. These pores help to release cytochrome c to the cytosol, which later binds Apaf-1 protein [40]. The cytochrome c bound with Apaf-1 triggers a set of downstream protein cascade reactions that finally activate procaspase-9 to caspase-9. Caspase-9 further activates procaspase-3/-7 and -6 to caspase-3/-7/-6, which eventually cleaves the inhibitors of nucleases [41]. Activated nucleases such as poly (ADP-ribose) polymerase (PARP) enter the nucleus and cause DNA degradation leading to cell death. On the contrary, extrinsic pathway signals originate from outside the cells. Upon binding of the extrinsic signal molecule ligands to any of the death receptors such as Fas, TNF*α*R, DR3, DR4, and DR5, the death domain receptor inside the cell is activated and induces the death-induced signaling cascade (DISC) due to interactions between multiple death receptors that are closely located to one another. Caspase-8, which plays a vital role in the extrinsic apoptotic pathway, is first activated. Caspase-8 activates procaspase-3/-7/-6 to caspase-3/-7/-6, which finally activates nucleases to degrade DNA. Although caspase-8 is a major protease of the extrinsic apoptotic pathway, it is also involved in the intrinsic apoptotic pathway by activating BID protein to tBID, which activates the Bcl-2 family members. BAX and BAK proteins are responsible for making holes in the mitochondrial membrane, triggering the intrinsic apoptotic pathway, as mentioned previously. Caspase-3 is regarded as the main executioner of caspase, and caspase-7 is responsible for cell detachment as a supportive role in apoptosis [42]. MCF-7 cells that we used here do not exist caspase-3 gene as the cells were originated from a breast cancer tissue without the caspase-3 protein-coding gene [43]. Therefore, caspase activity detected in the treated MCF-7 cells may be involved mainly with caspase-7 and other associated proteases of subfamily member CED-3. Further investigations are required to carry out in the future to identify other caspases involved in inducing apoptosis of such MCF-7 cells deficient in caspase-3. Time-dependent treatments are also recommended so that treatment duration required to attain the maximum number of early apoptotic cell population can be identified before treated cells enter the late apoptosis or secondary necrosis phase after 24 h of treatment.

Targeting cell cycle arrest is an attractive therapeutic property for anticancer compounds. In fact, interferon-alpha, an effective anticancer agent clinically used to treat liver cancer, also induces cell cycle arrest at the S and G2/M phases [44]. Other anticancer agents that induce cell cycle arrest at both the S and G2/M phases are selenomethionine (SeMet), gatifloxacin, and 7-hydroxystauro-sporine [45–47]. MDA-MB-231 cells treated with 4.5 *μ*M (lower than IC_50_), 8.5 *μ*M (IC_50_), or 12.5 *μ*M (higher than IC_50_) of compound 3F1 showed higher percentages in the S phase (18.08 ± 1.66%, 17.88 ± 0.33%, and 21.33 ± 1.49%) and G2/M phase (30.29 ± 6.17%, 35.58 ± 4.35%, and 36.59 ± 2.82%), respectively, compared with control cells in the S phase (11.66 ± 3.94%) and G2/M phase (24.15 ± 6.04%). MCF-7 cells treated with 3.5 *μ*M (lower than IC_50_), 6.5 *μ*M (IC_50_), or 9.5 *μ*M (higher than IC_50_) of compound 3F1 also showed higher percentages in the S phase (18.10 ± 1.80%, 20.74 ± 3.24%, and 22.19 ± 2.38%) and G2/M phase (30.17 ± 1.90%, 32.91 ± 3.32%, and 35.86 ± 2.76%), respectively, compared with MCF-7 control cells in the S phase (16.94 ± 0.36) and G2/M phase (26.86 ± 1.13%). Along with accumulation at the S and G2/M phases, a concomitant decrease in the percentage of G0/G1 phase cells was observed in the treated MDA-MB-231 (from the control at 64.20 ± 7.38% to 51.63 ± 7.58%, 46.53 ± 4.24%, and 42.07 ± 3.74%) and MCF-7 (from the control at 56.19 ± 0.98% to 51.84 ± 0.69%, 46.35 ± 0.19%, and 41.95 ± 0.85%) cells, respectively. Therefore, based on these consistent findings, we suggest that the novel gold compound 3F1 induced cell cycle arrest at both the S and G2/M phases.

Malignant cells grow uncontrollably due to disruption of cell cycle regulations by overexpression of cyclins and failure of cyclin-dependent kinase (CDK) inhibitor expression. Furthermore, incompetent checkpoint control also causes the division of genetically damaged cells resulting in the emergence of cancer cells. Both CDK4-cyclin D and CDK6-cyclin D are responsible for the G1 phase, whereas CDK2-cyclin A is responsible for the S phase. The G2/M phase is controlled by CDK1-cyclin A and CDK1-cyclin B, respectively [48]. For more detailed information regarding the cell cycle arrest induced by compound 3F1, it is necessary to determine the duration of each phase in the cell cycle to identify and quantify the respective CDKs with their associated cyclin proteins and CDK inhibitors specific to each phase of the cell cycle in tumor cells [49–51]. Our study suffers from some limitations. Normal breast cancer cells, e.g., MCF-10/-10A, should have also been tested with the compound 3F1 in addition to H9c2 cells. However, in other similar studies, the same H9c2 cell type was the only cells that had been tested without using normal breast too [52–55]. Normal breast cells may have more tolerance to the compound 3F1 than H9c2 cardiomyoblasts. Moreover, other caspases activities and the length of each cell cycle phase could have been carried out to present comprehensive findings.

## 5. Conclusions

Among newly synthesized gold (I) compounds, 3F1, 3F3, and the ligand 3FL, the compound 3F1 (triphenylphosphanegold(I) O-methyl-N-3-fluorophenyl thiocarbamate) demonstrated significant inhibition of the *in vitro* growth of triple-negative breast cancer cells (MDA-MB-231), estrogen receptor-positive breast cancer cells (MCF-7), BCA, BCSC-P and BCSC. The compound 3F1 induced apoptosis in both MDA-MB-231 and MCF-7 cells. Execution of MDA-MB-231 cell death was via activation of the caspase 3/7 pathway that is responsible for both extrinsic (death receptors and ligands) and intrinsic (mitochondrial-initiated) caspase-dependent apoptosis. IC_50_ doses of the 3F1 compound were approximately at least 6-fold lower than those of the commonly used anticancer compound, cisplatin (CDDP). Furthermore, 3F1 induced cell cycle arrest of treated MDA-MB-231 and MCF-7 cells at the S and G2/M phases. In summary, the compound 3F1, Ph_3_PAu[SC(OMe)=NC_6_H_4_F-3], is a potential anticancer compound, and findings from this study may help contribute to the development of a new promising anticancer therapeutic drug against breast cancer. *In vivo* future experiments are appropriate to find out further if the compound 3F1 demonstrates anticancer properties in the body system of breast cancer models and conduct more *in vitro* assays with other type of cancer cells such as head and neck cancer, lung cancer, prostate cancer, and ovarian cancer for toxicity and spectrum of anticancer properties of the compound 3F1.

## Figures and Tables

**Table 1:** IC_50_ of gold (I) compounds 3F1, 3F3, and 3FL, and CDDP (cisplatin) at 24 h.

Cell lines	IC_50_ (*μ*M)^a^			
	3F1	3F3	3FL	CDDP
MDA-MB-231	8.44 ± 0.81	>60	>80	50.20 ± 8.48
MCF-7	6.17 ± 0.66	>80	>80	33.13 ± 7.33
H9c2	13.67 ± 1.58	NA	NA	236.00 ± 96.52
BCA	5.77 ± 0.91	NA	NA	33.30 ± 9.22
BCSC-P	4.52 ± 0.54	NA	NA	89.11 ± 18.35
BCSC	10.17 ± 0.42	NA	NA	123.80 ± 21.07

^a^Concentration of the respective compound required for 50% inhibition of cell viability determined by the MTT assay. The results are reported as the mean ± SD of three independent experiments.

**Table 2:** IC_50_ of compound 3F1 at 24 h (MTT assay versus RTCA).

Cells lines	IC_50_ (*μ*M)^a^	
	MTT	RTCA
MDA-MB-231	8.44 ± 0.81	8.85 ± 0.38
MCF-7	6.17 ± 0.66	6.10 ± 0.28
H9c2	13.67 ± 1.58	15.30 ± 3.11

^a^Concentration of compound 3F1 required for 50% inhibition of cell viability determined by the MTT assay and RTCA. The results are reported as the mean ± SD of three independent experiments for MTT assays and two independent experiments for RTCA..

**Table 3:** Luna AO/PI cell viability assay.

Particulars	AO/PI cell viability	
	Control (48 h)	Treated (48 h) (24 h each before and after treatment)
MDA-MB-231 Seeded cells	400,000 cells/mL × 4 mL (1.6 ×10^6^ cells)	
Live cells	1.42 × 10^6^ cells/mL × 5 mL (7.10 × 10^6^ cells)	4.16 × 10^5^ cells/mL × 5 mL (2.08 × 10^6^ cells)
Viability % after treatment	29.29%	

MCF-7 Seeded cells	400,000 cells/mL × 4 mL = 1.6 × 10^6^ cells	
Live cells	8.46 × 10^5^ cells/mL × 5 mL (4.23 × 10^6^ cells)	5.45 × 10^5^ cells/mL × 5 mL (2.73 × 10^6^ cells)
Viability % after treatment	64.54%	

## Data Availability

The data that support the findings of this study are available from the corresponding author upon pragmatic request.

## Abbreviations

ATCC: American Type Culture Collection
BCSC: Breast cancer stem cells
HBSS: Hanks' balanced salt solution
DMEM: Dulbecco's modified eagle medium
FBS: Fetal bovine serum
EDTA: Ethylenediaminetetraacetic acid
DMEM/F12: Dulbecco's modified eagle medium/nutrient mixture F-12
MTT: 3-(4,5-Dimethylthiazol-2-yl)-2,5-diphenyltetrazolium bromide
CDDP: Cis-diamineplatinum (II) dichloride
DMSO: Dimethylsulfoxide
Bcl2: B-cell leukemia and lymphoma
BAX: Bcl2-associated X
HER2: Human epithelial growth factor receptor 2
ssDNA: Single-stranded DNA
CDK: Cyclin-dependent kinase
DISC: Death-induced signaling cascade
AO: Acridine orange
PI: Propidium iodide
PE: Phycoerythrin
7-AAD: 7-Amino-actinomycin-D
RNase: Ribonuclease
PS: Phosphatidylserine
RTCA: Real-time cell analysis.
